# Asynchronous Teleophthalmology for Monitoring Glaucoma Patients in a Rural German Region: A Retrospective Observational Pilot Study

**DOI:** 10.7759/cureus.24210

**Published:** 2022-04-17

**Authors:** Lukas Bisorca-Gassendorf, Simo Murovski, Henrike Julich-Härtel, Annekatrin Rickmann, Julie E Szabo, Mariya Erokhina, Martin Wenzel, Kai Januschowski

**Affiliations:** 1 Ophthalmology, Eye Clinic Petrisberg, Trier, DEU; 2 Ophthalmology, Eye Centre Erzgebirge, Zschopau, DEU; 3 Ophthalmology, Eye Clinic Sulzbach, Knappschaft Hospital Saar, Sulzbach/Saar, DEU; 4 Ophthalmology, University Eye Hospital Tübingen, Tübingen, DEU

**Keywords:** glaucoma practice, teleglaucoma, teleophthalmology, telemedicine, telehealth

## Abstract

Background: Coronavirus disease 2019 (COVID-19) has created an escalating need for limiting in-person examination and potential viral exposure. Under these circumstances, teleophthalmology allows ophthalmologists to continue providing care to patients while ensuring their safety and that of the medical staff.

Objective: The primary objective of this study was to assess patient satisfaction with an asynchronous teleconsultation for glaucoma patients in a rural German area. Secondary endpoints were patient adherence and the need to change the therapeutic regime.

Methods: This retrospective, observational, and monocentric study included 50 patients diagnosed with primary open-angle glaucoma (n = 49) and ocular hypertension (n = 1) requiring medication to lower intraocular pressure (IOP). Only patients with well-controlled diseases were included, and a brief questionnaire was evaluated, which was completed one year after the baseline visit. Best-corrected visual acuity (BCVA), IOP measurements, visual fields, optical coherence tomography images of the optic nerve head, ultra-widefield photographs of the fundus, and photographs of the anterior segment of the eye were taken at each visit by an experienced optometrist.

Results: Of the 50 patients included, the mean number of follow-up visits in this observation period was 4.4. No patient was lost to follow-up, and there were a total of nine missed follow-up visits (but not lost to follow-up). No patients required a change in their treatment regime during the observational period. Regarding patient-focused assessment, the majority of patients were satisfied or very satisfied with teleconsultation in general.

Conclusion: Asynchronous teleophthalmology is a promising option and effective means to monitor glaucoma patients. The majority of teleophthalmology patients were satisfied with their teleconsultation and adhered to the follow-up schedule. However, prospective trials with a larger number of patients and a more focused examination on specific patient populations are required. Further trials should also focus on the aspect of cost-effectiveness.

## Introduction

Glaucoma is the most common cause of irreversible blindness worldwide [1]. This group of eye diseases leads to a progressive constriction of the visual field owing to optic nerve damage, and even mild forms can cause impairment in reading or walking with an increased risk of accidents. The majority of people living with glaucoma remain undiagnosed [2]. Existing treatment options only aim at slowing the progression of the disease, necessitating the constant monitoring of affected patients. Patients do not only suffer from vision loss but are more likely to show earlier and more severe physical and psychological decline [2-6]. Many glaucoma patients have only limited or no access to an ophthalmologist and could potentially benefit from innovative care concepts that facilitate screening and regular follow-up visits, but not necessarily with direct doctor-patient contact at the moment of the examination. The latest developments in digital and remote screening procedures are promising [7]; however, a recent meta-analysis has shown that screening, in particular, is inferior to physical examination. Nevertheless, poor accessibility and lack of specialized ophthalmologists in many areas and countries are factors that are not likely to improve in the near future; many developed countries also face the challenges of a growing older population with a higher incidence of glaucoma, making innovative solutions more important than ever. One possible solution to address the discrepancy between promising telemedical options and the unmet need for telemedical screening of glaucoma patients is to strengthen the teleophthalmological aspect of uncomplicated follow-up visits of patients after they have been diagnosed with glaucoma.

This option seems reasonable because ophthalmologists usually base their clinical decisions about the course of glaucomatous patients on three measures: morphometric changes, functional changes, and intraocular pressure (IOP) [8]. All three examinations could potentially be delegated to medical technicians under an ophthalmologist’s supervision, with hypothetically only one minor exception: IOP measurements with Goldmann applanation tonometry, which should be performed in critical cases. Therefore, most of the uncomplicated glaucoma follow-up patients can be monitored using other methods [9]. In a regular follow-up visit, an in-person conversation and assessment of the patient by an ophthalmologist would be optimal; however, most patients do not require a change in treatment strategy [10] and could thus benefit from asynchronous teleophthalmology, which delivers the benefits of facilitated access to examinations, less waiting time, and regular follow-ups. In effect, specialized ophthalmologists could devote more time to urgent patients and handle remote areas.

Currently, two models of teleophthalmology are used: synchronous and asynchronous. Synchronous teleophthalmology is characterized by a teleconsultation with a direct, live patient-doctor interaction via voice-only or voice and video, whereas asynchronous teleophthalmology adopts an alternative model in which investigations are carried out by a specialized medical assistant without the presence of a doctor at the time of the investigation. After all required investigations have been undertaken, the patient can leave the clinic, yielding shorter waiting times. Afterward, the doctor revises the studies within a defined time and then sends a written report to the patient.

The primary objective of this study was to assess patient satisfaction with an asynchronous teleconsultation for glaucoma patients in a rural German area. Secondary endpoints were patient adherence and the need to change the therapeutic regime. A patient questionnaire about general aspects of teleconsultation was undertaken on 200 patients with different ocular pathologies. The study only included patients who have been thoroughly examined by a board-accredited specialist in ophthalmology and were diagnosed with primary open-angle glaucoma or ocular hypertension, according to the guidelines of the European Glaucoma Society. This work was presented at the 94th Meeting of the Association of Rhine-Main Ophthalmologists in Koblenz, Germany on October 30, 2021.

## Materials and methods

This retrospective observational study included patients with open-angle glaucoma or ocular hypertension who were diagnosed and under constant follow-up at the Eye Center Zschopau, Germany. All patients were over 18 years old and were informed about the nature of teleophthalmological monitoring beforehand. The study was approved by the Ethics Committee of the Rhineland-Palatinate Medical Association and adhered to the tenets of the Declaration of Helsinki. This pilot project was supported by local health insurance companies and the medical association of Saxony/Germany.

Baseline data, such as age, gender, diagnosis, number of glaucoma medications, best-corrected visual acuity (BCVA), slit-lamp examination, and dilated fundus examination results, were obtained from all patients or were available from the electronic records. Only patients with a stable situation (i.e., no progression in optical coherence tomography (OCT) of the optic nerve head (ONH), no progressive visual-field defects, and IOP of under 25 mmHg) were included in the remote monitoring group. Patients with a certain risk for angle-closure were not included. BCVA, IOP measurements (Tonoref II, Nidek, Aichi, Japan; Ocular Response Analyzer G3, Reichert Technologies, Depew, USA), OCT images of the ONH (Heidelberg Engineering, Heidelberg, Germany), visual fields (Octopus 900, Haag-Streit, Köniz, Switzerland), ultra-widefield scanning laser ophthalmoscopy images of the fundus (Daytona, Optos, Marlborough, USA), and anterior segment photographs were taken during each visit (including baseline) by one of four experienced optometrists. Two of the optometrists had also received Good Clinical Practice (GCP) certificates in November 2020 and all employees were trained internally on the proper execution of the above-named examinations. The first consultation also involved contact with an ophthalmologist, whereas subsequent visits were performed solely using asynchronous teleophthalmology. Follow-up visits were scheduled every three to six months. After each visit, a written report of the teleconsultation was issued and mailed to the patient. All performed examinations (i.e., BCVA, IOP, and special diagnostics such as OCT) were connected to the medical practice management software (FIDUS, Arztservice Wente GmbH, Darmstadt, Germany) via DICOM (Digital Imaging and Communications in Medicine) and could be viewed by the responsible ophthalmologist. Generated reports were stored in the medical practice management software and transmitted to the patient.

Patient satisfaction was assessed using a questionnaire that included six questions on various aspects, such as the patient’s satisfaction regarding scheduling appointments or whether teleconsultation is considered a good substitute for in-person consultations with a doctor. The survey was conducted in German after the one-year control and each question could only be answered with one of the following choices: strongly agree, agree, neutral, or disagree. Table 1 shows an English translation of the questionnaire.

**Table 1:** English translation of the questionnaire

Question #	
Q1	Did you feel well informed during your informative discussion about teleconsultation with the doctor or staff?
Q2	Are you satisfied with how your questions and/or concerns were addressed during your appointment?
Q3	Do you feel that teleconsultation, as you have experienced it, is a good substitute for a consultation with in-person doctor contact?
Q4	Are you satisfied with the outcome (see report) of your appointment?
Q5	Do you feel that the report is understandable?
Q6	Are you satisfied with the scheduling of appointments for the consultation?

## Results

We analyzed 50 patients retrospectively. The visits took place from April 2019 to March 2021, while the mean period of observation was 18.2 months (range: 16-21 months). A total of 49 patients suffered from primary open-angle glaucoma, while one patient was diagnosed with ocular hypertension without requiring IOP-lowering medication. The mean age was 69.9 years, with an age range of 49-92 years. We included 28 female and 22 male patients. The mean IOP throughout all visits of all patients was 14.9 mmHg. Patients required an average of 1.4 IOP-lowering medications. An overview of the clinical characteristics of the study population is given in Table 2.

**Table 2:** Overview of the clinical characteristics of the study population

Characteristics	Values
Age (mean)	69.9 years
Age (range)	49-92 years
Female sex	N = 28
Male sex	N = 22
Observation period (mean)	18.2 months
Observation period (range)	16-21 months
Mean number of visits	4.4 per patient
Mean intraocular pressure (IOP)	14.9 mmHg
Mean number of IOP-lowering drugs	1.4 per patient

Of the 50 patients included, the mean number of follow-up visits in this observation period was 4.4. No patient was lost to follow-up. The total number of missed follow-up visits (but not lost to follow-up) was nine. Regarding frequency, nine patients had only three visits, 24 patients had four visits, and 17 patients had five or more visits. No patients required a change in their treatment regime during the observational period.

**Figure 1:**
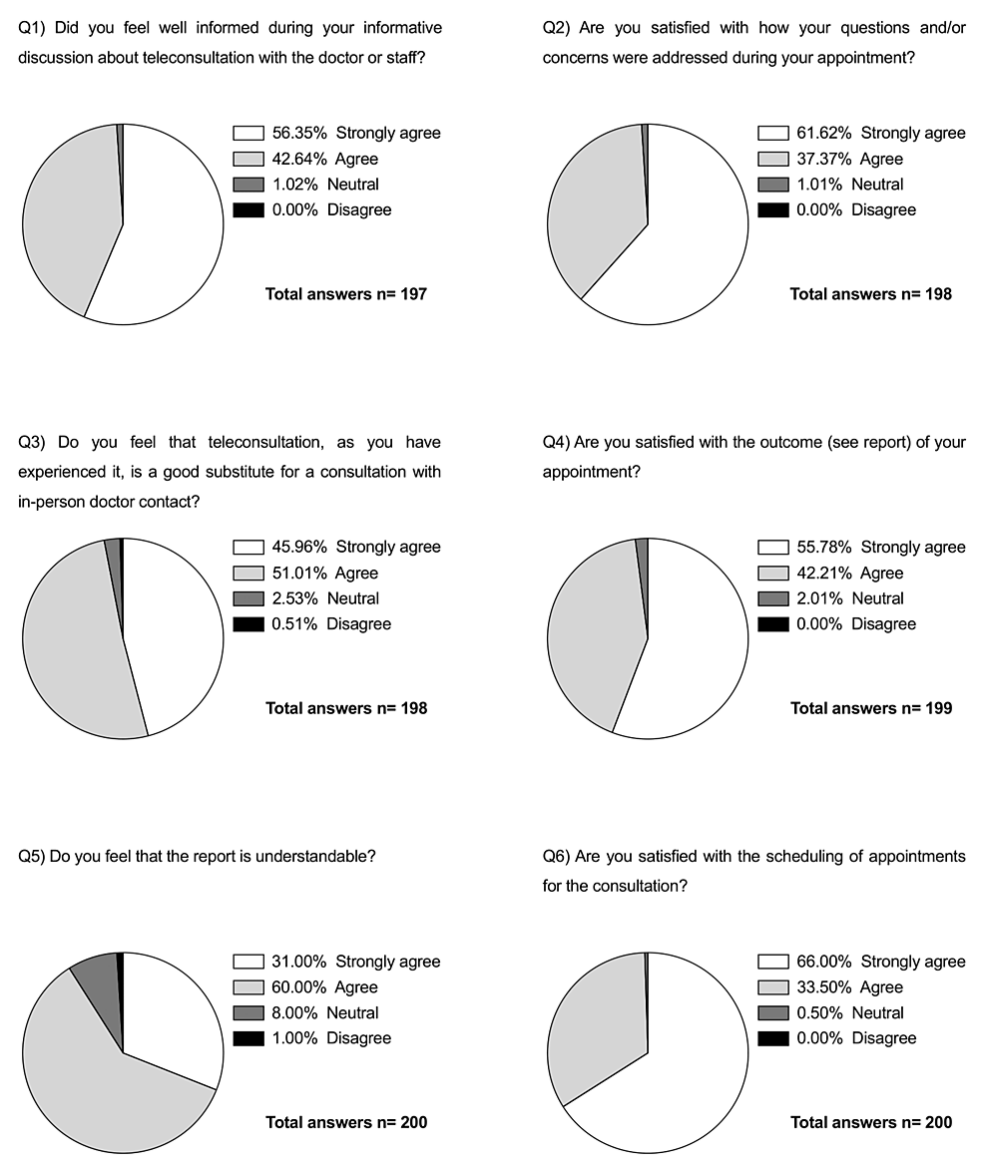
Circle graphs of patients’ responses to the survey conducted with a total of six questions

Regarding patient-focused assessment, the majority of patients were either satisfied or very satisfied with teleconsultation in general. Figure 1 represents the patients' answers to the conducted survey in more detail. The total number of responses is higher than that of the included patients because the survey was conducted not only in glaucoma patients but also in patients with other eye diseases, such as diabetic retinopathy or age-related macular degeneration. Data protection requirements prohibited specifying the ocular pathology; therefore, it had to be considered a confounding factor. Since some questions were not answered correctly and were excluded from the analysis (missing answers or more than one marked answer), the total number of answers varies between 197 and 200.

Regarding patient-doctor interaction, all patients received a written report of the examination within five days following the examination, stating potential treatment strategies and dates of follow-up visits. If patients had additional questions or concerns, they raised them with our trained optometrists and were subsequently contacted by a physician if needed. However, this was not often necessary.

## Discussion

In this study, we showed that the majority of patients were satisfied with their teleconsultation and adhered to the follow-up schedule. Most patients accepted this innovative approach in a rural area of Germany. Long-term treatment monitoring of glaucoma patients is as essential as it is tedious for the patient and the treating ophthalmologist. Teleophthalmological solutions are an attractive tool for monitoring stable patients after initiation of treatment and addressing the current dilemma, in which screening is still suboptimal and access to specialized care is scarce, but technical options for follow-up are promising. These aspects are even more important when considering finances [11]. Recently, teleophthalmology has received significantly more attention and is developing rapidly owing to the coronavirus disease 2019 (COVID-19) pandemic, which has increased the demand for telemedicine in general, owing to its ability to reduce in-person examination and possible virus exposure [12-14]. Our findings show that this increased need can be addressed effectively, and treatment monitoring is possible without the need for a synchronized patient-doctor interaction. Probably the most important finding is that patients were very satisfied with this specific asynchronous approach and did not miss follow-up visits during the first year. This level of adherence is better than that reported in the literature [15] and might be attributed to the fact that patients were specifically informed about the possibility of delayed reporting, yielding shorter waiting times without sacrificing medical accuracy. Similar findings were reported from a national survey on the acceptability and use of glaucoma clinics in the United Kingdom (UK). Based on these findings, Gunn et al. [16] concluded that patients’ acceptability was comparable to that of standard care.

Our results are reflected in the literature [17] and stress the great potential of innovative approaches to meet the growing challenges. In the UK, this concept has been evaluated and labeled as “teleglaucoma,” but mixed results have been found for long-term treatment monitoring [18]. Although our results are encouraging, it should be mentioned that, from a patient’s perspective, an encounter with a trusted doctor would be optimal. This is another important factor that might contribute to the adherence level demonstrated in this study [15]. Nevertheless, one limitation of the survey is that it was not conducted only for patients with glaucoma but also included those with other pathologies. Unfortunately, the retrospective analysis of the anonymized questionnaires does not allow us to draw any conclusions about the underlying disease of each patient, but we plan to consider this point separately in our future work. Patient adherence toward the application of IOP-lowering drugs was not assessed in this study. Excellent patient adherence regarding follow-up visits and good IOP-control might indicate a good overall adherence but no direct data were generated in this pilot study setting. Moreover, the lack of any control group has to be highlighted.

To minimize direct contact in a pandemic situation, it would have been optimal to reduce interaction not only between patients and doctors but also between patients and optometrists. Nevertheless, all staff and patients were equipped with FFP2 masks from the onset of the COVID-19 pandemic, and examinations were precisely timed, limiting patient-to-patient contact in a virtually empty waiting room.

It should be noted that this pilot study only examines a mean period of 18 months, whereas glaucoma patients need to be monitored over longer periods of time. Further studies should not only include a larger number of patients but also compare the level of satisfaction to that of patients who received in-person consultation with a doctor and focus as well on the aspect of cost-effectiveness.

In summary, telemedical approaches are well tolerated by the vast majority of patients and are an attractive alternative to the standard of care in areas where access to ophthalmologists is limited.

## Conclusions

Asynchronous teleophthalmology is a promising option and effective means to monitor glaucoma patients. The majority of teleophthalmologic patients were satisfied with their teleconsultation and adhered to the follow-up schedule. However, prospective trials with a larger number of patients and a more focused examination on specific patient populations are required. Further trials should also focus on the aspect of cost-effectiveness.
